# Antibiofilm Activity and Mechanism of Action of the Disinfectant Chloramine T on *Candida* spp., and Its Toxicity against Human Cells

**DOI:** 10.3390/molecules22091527

**Published:** 2017-09-17

**Authors:** Gabriela Lacet Silva Ferreira, Pedro Luiz Rosalen, Larissa Rangel Peixoto, Ana Luiza Alves de Lima Pérez, Fabíola Galbiatti de Carvalho Carlo, Lúcio Roberto Cançado Castellano, Jefferson Muniz de Lima, Irlan Almeida Freires, Edeltrudes de Oliveira Lima, Ricardo Dias de Castro

**Affiliations:** 1 Graduate Program in Dentistry, School of Dentistry, Universidade Federal da Paraíba (UFPB), João Pessoa PB 58051900, Brazil; gabrielalacet@yahoo.com.br (G.L.S.F.); larissarngl@gmail.com (L.R.P.); analuiza_perez@yahoo.com.br (A.L.A.d.L.P.); fabigalbi@yahoo.com.br (F.G.d.C.C.); luciocastellano@gmail.com (L.R.C.C.); jefferson.idalino@gmail.com (J.M.d.L.); edelolima@yahoo.com.br (E.d.O.L.); 2 Department of Physiological Sciences, Piracicaba Dental School, University of Campinas (UNICAMP), Piracicaba SP 13414903, Brazil; rosalen@fop.unicamp.br; 3 Department of Oral Biology, University of Florida College of Dentistry, Gainesville, FL 32610, USA; IFreires@dental.ufl.edu

**Keywords:** stomatitis, denture, denture cleansers, chloramines, candidiasis, oral, biofilms

## Abstract

We evaluated the antifungal and anti-biofilm activity, mechanism of action and cytotoxicity of chloramine T trihydrate (CAT) against *Candida* spp. The Minimum Inhibitory and Fungicidal Concentrations (MIC/MFC) of CAT were determined. Changes in CAT-treated *C. albicans* growth kinetics and micromorphology were evaluated, as well as the mechanism of action, and its effects on biofilm. Cytotoxicity was assessed by the hemolysis method. The data were analyzed by inferential statistics (*p* ≤ 0.05). CAT showed antifungal activity against all strains, with MIC values ranging between 1.38 and 5.54 mmol/L (MIC_75%_: 2.77 mmol/L). CAT demonstrated an immediate and sustained action on *C. albicans* growth kinetics, particularly at 2 × MIC. This compound likely acts on the cell wall and membrane permeability simultaneously and was found to cause changes in *C. albicans* micromorphology. Tha antibiofilm activity of CAT was similar to that of sodium hypochlorite (*p* > 0.05) against mature biofilms. CAT was more effective than NaOCl in reducing mature biofilm upon 1-min exposure at 2 × MIC (24 h) and 4 × MIC (48 h) (*p* < 0.05). Toxicological analysis revealed that CAT had hemolytic activity between 61 and 67.7% as compared to 100% by NaOCl. CAT has antifungal and anti-biofilm properties, probably acting on both cell wall and membrane permeability, and showed low toxicity in vitro.

## 1. Introduction

Denture stomatitis is an inflammatory and erythematous condition of the oral mucosa mostly commonly related to the use of complete upper dentures, with prevalence rates among denture wearers ranging from 15% to over 70% [1]. This disease is strongly associated with the presence of *Candida* spp. strains and may be triggered or aggravated by frequent trauma, poor denture adaptation, deficiency in salivary flow, smoking habits, poor oral hygiene, continuous denture use, and immunosuppression [1,2,3,4].

While oral candidiasis has been associated with the use of dentures, it has been well documented that *C. albicans* is the main causative agent of denture-related infections. However, other species such as *C. tropicalis*, *C. krusei* and *C. glabrata* have also been isolated from the mucosa and denture bases of patients and thus are thought to be important agents in the disease onset [2,5,6]. The fact that these *Candida* yeasts can be isolated from the mucosa does not necessarily indicate disease, but dentures can indeed be a favorable site for adherence and proliferation of microorganisms that are likely to cause disease in a susceptible host. 

Mechanical denture cleaning is the most commonly used method to prevent biofilm development on the acrylic surface of dentures, but the adjunctive use of chemicals is highly encouraged as a way to enhance cleaning effectiveness [7,8,9]. Sodium hypochlorite is frequently indicated as a denture cleaning solution [7,10]. Nevertheless, its undesirable characteristics, such as unpleasant residual odor and flavor, risk of mucosal burning, metal corrosion and acrylic resin color change [7,11,12], make it an unideal alternative. These limitations encourage the search for much needed chemical compounds that can effectively inhibit or eradicate denture biofilms, with fewer side effects.

**Figure 1:**
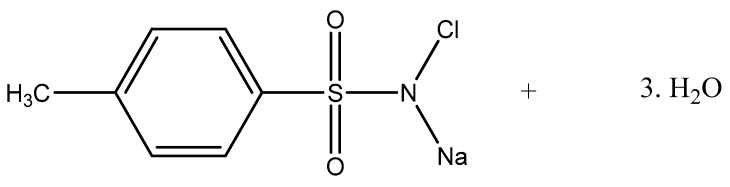
Chemical structure of Chloramine T trihydrate (C_7_H_7_ClNNaO_2_S·3H_2_O).

Chloramine T (CAT) is an active chlorine compound with the chemical formula C_7_H_7_ClNNaO_2_S·3H_2_0 (molecular weight: 281.68 g/mol) (Figure 1). This synthetic compound has proven antimicrobial activity as a result of its chlorination/oxidative power [13,14]. The stability [15] and antiseptic capacity [16,17] of CAT have been studied for many years. In dentistry, CAT has been used as a bactericide and disinfectant agent [18]. There are also studies in the literature investigating CAT-based dental creams suitable for denture cleaning [19,20]. However, little research has been carried out, to date, focused on the antifungal activity and mechanism of action of CAT, particularly against *Candida* spp. biofilms. Herein, we describe the antifungal activity, mechanism of action, and anti-biofilm potential of CAT on seven *Candida* strains, as well as its toxicity in vitro against human cells.

## 2. Results

### 2.1. Determination of the MIC and MFC Values

As seen in Table 1, CAT was tested for its ability to inhibit microbial growth of seven reference strains of *Candida* spp. The results demonstrated that CAT has antifungal activity against *C. albicans*, *C. tropicalis*, *C. krusei* and *C. glabrata*, with MIC and MFC values ranging between 0.69–5.54 mmol/L and 1.38–11.09 mmol/L, respectively. The concentration of 2.77 mmol/L was able to inhibit 75% of the tested strains (MIC_75_). Sodium hypochlorite showed MIC and MFC values ranging between 4.19 and 16.79 mmol/L. The MFC/MIC ratio indicated that CAT had predominantly fungicidal activity against the tested strains, similarly to sodium hypochlorite and nystatin.

### 2.2. Effects on C. Albicans Growth Kinetics

**Figure 2:**
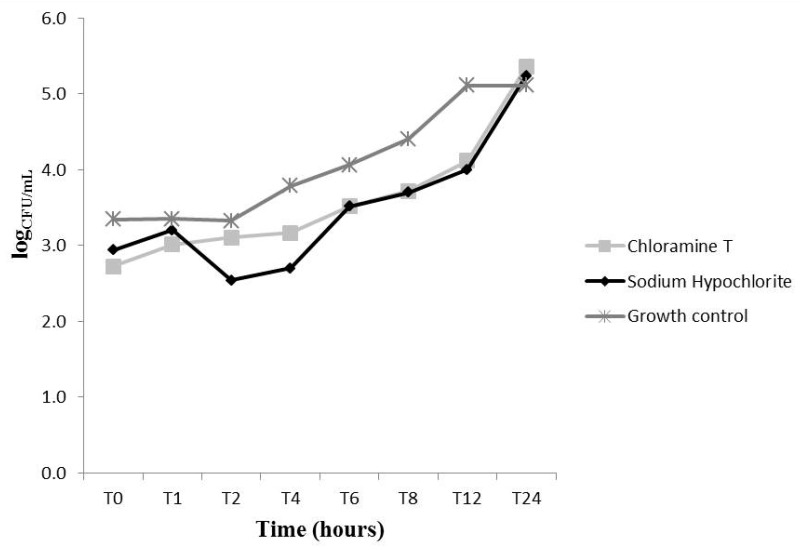
*Candida albicans* ATCC 60193 fungal growth kinetics upon treatment with chloramine T (2.77 mmol/L) and sodium hypochlorite (8.39 mmol/L) at their MIC concentrations.

**Figure 3:**
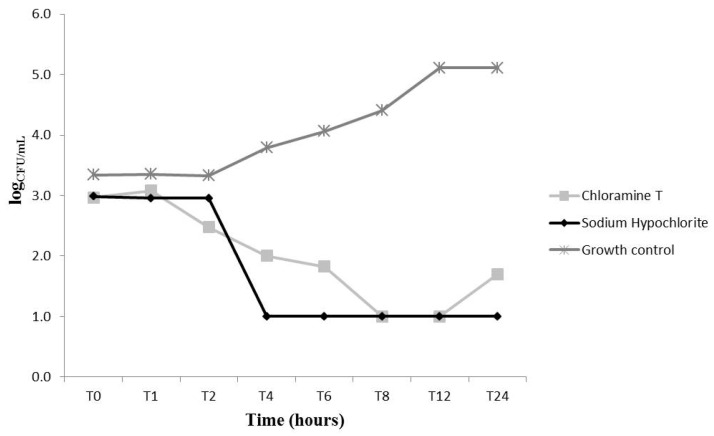
*Candida albicans* ATCC 60193 fungal growth kinetics upon treatment with chloramine T (5.54 mmol/L) and sodium hypochlorite (16.79 mmol/L) at 2 times their MIC concentrations.

**Figure 4:**
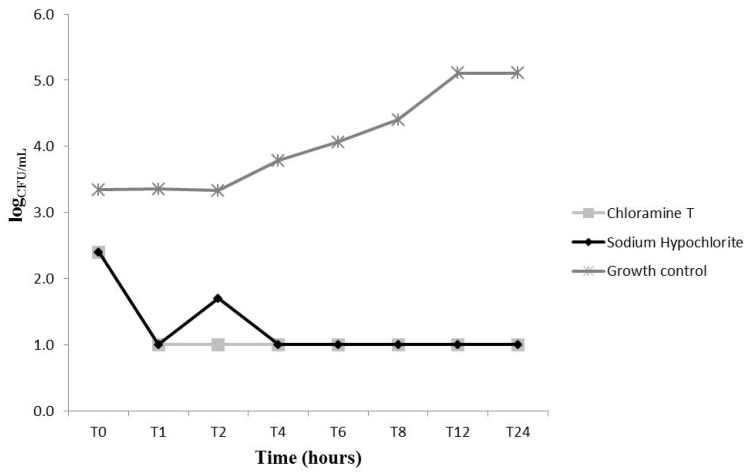
*Candida albicans* ATCC 60193 fungal growth kinetics upon treatment with chloramine T (11.09 mmol/L) and sodium hypochlorite (35.58 mmol/L) at 4 times their MIC concentrations.

We evaluated the fungal growth kinetics upon treatment with CAT and NaOCl at different concentrations (MIC, 2 × MIC and 4 × MIC) (Figure 2, Figure 3 and Figure 4). At MIC, CAT and NaOCl inhibited fungal growth at initial exposure (T0) as compared to the untreated group (*p* < 0.05). NaOCl had maximum inhibition after 8 (T8) and 12 (T12) hours of exposure, respectively (*p* < 0.05), while CAT did not exert significant effects over time at this concentration (Figure 2). Nevertheless, CAT and NaOCl at 2 × MIC had a significant effect on growth kinetics after 2 h (T2) of exposure, leading to complete growth inhibition after 6 hours (*p* < 0.05), with increased fungal growth after 24 hours (T24), indicating a loss of CAT substantivity after this period (Figure 3). These data provide information necessary to establish disinfection protocols. At 4 × MIC, CAT and NaOCl significantly inhibited fungal growth at all checkpoints as compared to the untreated group (*p* < 0.05) (Figure 4). There was no difference in the antifungal effectiveness of CAT and NaOCl when treated at 4 × MIC (*p* > 0.05).

### 2.3. Mechanism of Action of CAT

Herein, we studied two possible mechanisms of action of CAT in the yeast cell, namely: disturbance of cell wall biosynthesis or cell membrane permeability. As seen in Table 2, the results showed that CAT may act on *C. albicans* cell wall—as does caspofungin, used as a positive control—because the susceptibility of the strains to these agents was diminished in the of presence sorbitol, an osmotic protector. This means that higher MIC values were necessary to disrupt the protected cell wall as compared to the medium without sorbitol. This same trend was also observed for sodium hypochlorite. Furthermore, their MIC values also increased between 1 and 8 folds with addition of exogenous ergosterol (Table 3), which suggests that they bind with ergosterol and this could potentially lead to pore formation in the membrane and perturbation of ionic permeability.

### 2.4. Effect of CAT on C. albicans Micromorphology

**Figure 5:**
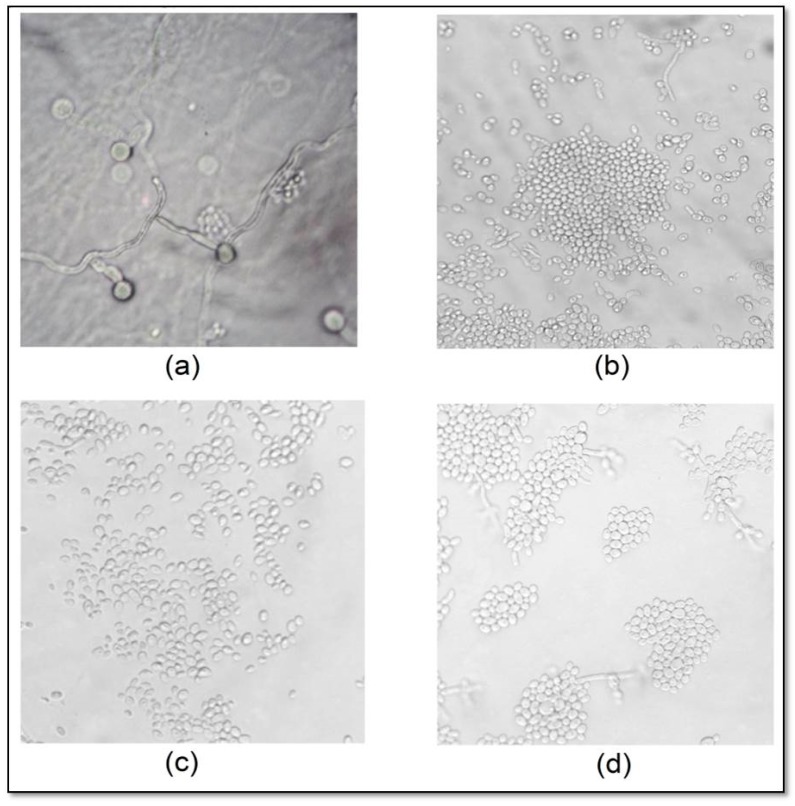
*Candida albicans* (CBS 562) micromorphology (40× magnification) in the absence (growth control) (**a**) and presence of chloramine T (**b**), sodium hypochlorite (**c**) and nystatin (**d**) at 2 × MIC.

Untreated *C. albicans* CBS 562 microculture showed the presence of abundant pseudohyphae, blastoconidia and chlamydospores, while treatment with CAT, sodium hypochlorite or nystatin led to changes in the filamentous form, which were directly proportional to the increase in concentration (Figure 5). CAT- and nystatin-treated cells showed impaired cellular development, with expression of rare pseudo-hyphae and absence of chlamydoconidia. Treatment with sodium hypochlorite at 16.79 and 33.58 mmol/L caused total inhibition pseudohyphae and chlamydospore formation. These results are illustrated in Figure 5.

### 2.5. Effects of CAT on the Adherence, Formation and Reduction of C. Albicans Mature Biofilms

The percentage of inhibition of CAT- or NaOCl-treated biofilms with different exposure times is shown in Table 4 and Table 5. In Table 4, it can be seen that NaOCl reduced C. *albicans* adherence significantly more at 2 × MIC (*p* < 0.05), but CAT demonstrated activity similar to that of NaOCl at all concentrations (*p* > 0.05). However, CAT was more effective (*p* < 0.05) at reducing *C. albicans* mature biofilm than NaOCl at 2 × MIC after three 1-min exposures for 24 h, and at 4 × MIC after six 1-min exposure over 48 h (Table 5). As for long exposure times (8 h), there was no difference between CAT and NaOCl at the concentrations tested. No statistically significant difference was found between the different concentrations of the same compound.

### 2.6. Cytotoxicity of CAT on Human Erythrocytes

All tested concentrations of CAT in the range between 88.75 and 2.70 mmol/L demonstrated hemolytic activity between 61% and 67.7%. Hypochlorite, used as a control, caused complete destruction of the erythrocytes immediately after being added to the wells, which was considered as parameter of 100% hemolytic activity. Saline (0.9% NaCl), used as a negative control, did not cause hemolysis, as expected.

## 3. Discussion

Although CAT has been studied for many years, there is still a lack of evidence regarding its use in dentistry as an antimicrobial agent [19,20]. This is the first study to evaluate the antifungal activity of CAT on *C. albicans*, *C. tropicalis*, *C. krusei* and *C. glabrata* strains as well as its effects on yeast growth kinetics and micromorphology. In addition, we further elucidated the mechanism of action of CAT and its anti-biofilm activity at different exposure times.

A study evaluating the fungicidal activity of CAT (0.35 mmol/L) on a clinical isolate of *Aspergillus fumigatus* did not find positive results [21]. As seen in our study, concentrations higher than the one tested by the authors are needed to significantly affect *Candida* spp. strains. The literature shows that CAT is also effective against bacterial strains (*Escherichia coli*, *Enterococcus faecalis*, *Staphylococcus aureus*, *Pseudomonas aeruginosa*, *Klebsiella pneumoniae*, *Proteus mirabilis*, *Enterobacter cloacae*, *Staphylococcus epidermidis*, and *Serratia marcescens*), with MIC values ranging between 1.77 and 7.10 mmol/L, which are similar to those found in our study [22].

A previous study described the death kinetics of *C. albicans* upon treatment with CAT. The authors found that at 3.55 mmol/L, CAT caused a 2-log_10_ reduction in CFU/mL upon 30 min exposure, while at 0.35 mmol/L this reduction was achieved after 1 h [13]. Herein, the lowest CAT concentration tested (2.77 mmol/L) was able to significantly reduce (*p* < 0.05) the number of yeasts immediately after addition of CAT (T0), but this concentration was not able to reduce the log_10_ level over time. At the concentration of 5.54 mmol/L (2 × MIC), CAT decreased the number of CFU/mL after 6 h, and at 4 × MIC (11.09 mmol/L) it caused a complete killing of yeast cells after 1 h.

Some chemical characteristics of CAT, such as its efficient chlorination and oxidation of microorganisms [23] as well as its high stability and ability to maintain chlorine levels for a prolonged time [15], may explain, respectively, the rapid antifungal effect at T0 and the prolonged action of CAT that caused significant inhibition of fungal growth up to 12 h at all tested concentrations. According to the literature, active chlorine compounds act primarily through the chlorination of the extracellular protein matrix of microorganisms, forming a chlorine layer. CAT has highly reactive compounds and a high capacity for chlorine layer formation that has an immediate destructive impact on the microbial surface [23]. The results of the present study are supported by these findings, because the MIC of CAT increased in the presence of both sorbitol and exogenous ergosterol, suggesting a simultaneous action on the cell membrane and cell wall.

The study of *C. albicans* micromorphology in the presence of antifungal agents is important, because fungal morphology is one of the several factors that affect its virulence and pathogenicity. The formation of pseudohyphae and hyphae causes tissue invasion by enabling the cell to exert a mechanical force favoring tissue penetration. In addition, chlamydospores are also considered to be resistance structures [24,25]. CAT reduced the formation of pseudohyphae and inhibited the formation of chlamydospores at all tested concentrations, while sodium hypochlorite completely inhibited the presence of filaments and chlamydospores at higher concentrations. This result confirms the antifungal activity of CAT observed in the other assays and indicates that CAT may induce yeast cells to form only blastoconidia, which are considered less virulent structures.

Taking into account the adherence and penetration capacity of *C. albicans* to the acrylic resin of dentures and the fact that hyphae lead to formation of thicker, more resistant biofilms [26], the results presented herein support the indication of CAT as a solution for denture disinfection. In denture stomatitis, the biofilm formed on the denture merits attention because disease can recur after drug treatment, in case there is remaining biofilm adhered to the material [26,27]. We found that CAT has anti-biofilm activity similar to that of sodium hypochlorite regarding inhibition of initial adherence and formation of mature biofilms. 

Substances indicated for denture disinfection can be harmful and thus should not be in contact with the patient’s mucosal tissues. For this reason, it is recommended to thoroughly wash the denture after disinfection. We evaluated the CAT cytotoxicity using the hemolysis method, which can be considered a preliminary, although meaningful, assay to evaluate the characteristics of test substances. All the CAT concentrations tested, either higher or lower than its MIC, showed hemolytic activity in the order of 60%, with no concentration-dependent effects. Sodium hypochlorite caused complete hemolysis immediately upon contact with the erythrocytes, confirming its high cytotoxicity. According to the literature [28], CAT did not cause visible cellular effects on a human cell line of squamous cell carcinoma at concentrations between 1 and 0.035 mol/mL after 30 min exposure. At higher concentrations (0.35, 3.55 and 35.50 mol/mL), it led to changes in cell morphology after 30 min, and caused complete fragmentation after 24 h.

The results of our study provide important knowledge about the inhibitory effects of CAT on fungal growth kinetics, its mode of action, effects on fungal micromorphology and anti-biofilm activity. CAT showed antibiofilm activity better than that of NaOCl in short contact times (1 min). Collectively, our findings demonstrate the good potential of CAT as a denture disinfectant.

Chloramine T showed fungicidal activity against *Candida* spp., possibly affecting both cell wall and membrane permeability. CAT was found to have deleterious effects against *C. albicans* micromorphology and anti-biofilm activity. A preliminary toxicological assay showed that it has high hemolytic activity, albeit lower than that of sodium hypochlorite. Further tests are needed in the field of toxicology, and also testing the anti-biofilm activity using a multi-species model, as well as the effect of CAT on the properties of denture components.

## 4. Materials and Methods

### 4.1. Microorganisms

Reference strains of *Candida* spp. were obtained from the American Type Culture Collection (ATCC, Rockville, MD, USA)—*C. albicans* ATCC 60193, *C. tropicalis* ATCC 750 and *C. krusei* ATCC 3413; from the Central Bureau voor Schimmelcultures (CBS, Baarn and Delft, The Netherlands, Holland)—*C. albicans* CBS 562, *C. tropicalis* CBS 94 and *C. krusei* CBS 73; and from the IZ collection (Instituto Zimotécnico at ESALQ/USP, Campinas, São Paulo, Brazil)—*C. glabrata* IZ 07.

### 4.2. Determination of the Minimum Inhibitory and Fungicidal Concentration (MIC)

The MIC of chloramine T trihydrate (INLAB-CAS 7080-50-4, São Paulo, Brazil) was determined using the microdilution technique according to the protocol proposed by the Clinical and Laboratory Standards Institute (CLSI) [29]. Briefly, an initial volume of Sabouraud Dextrose Broth (SDB, KASVI, Curitiba, Brazil) was added to 96-well U-bottom microdilution plates. Then CAT solution starting at 88.75 mmol/L was placed into the first well of the plate and serially diluted. Nystatin (Sigma-Aldrich, São Paulo, Brazil) was used as an internal control to monitor strain susceptibility and sodium hypochlorite (Sigma-Aldrich) was used as positive control as denture disinfectant. Microbial viability and sterility controls were also checked. Fungal inoculum was added to each well at a final concentration of 2.5 × 10^3^ CFU/mL. The plates were incubated at 37 °C for 24 h. Aliquots of 50 µL of TCT (2,3,5-triphenyl tetrazolium chloride) dye were added to the wells in order to confirm the presence of viable microorganisms, and then incubated again for 24 h. The MIC was defined as the lowest concentration of CAT that inhibited visible fungal growth.

Aliquots from the wells corresponding to the MIC and higher concentrations were subcultured onto Sabouraud Dextrose Agar (SDA, KASVI) plates and incubated at 37 °C for 24 h. The MFC was defined as the lowest concentration of CAT that inhibited visible growth on the solid medium. The MFC/MIC ratio was calculated to determine whether the compound had fungistatic (MFC/MIC ≥ 4) or fungicidal (MFC/MIC < 4) activity [30].

### 4.3. Effects of CAT on Candida spp. Growth Kinetics

The synthetic compound CAT was tested for its antifungal effects against *C. albicans* ATCC 60193 growth kinetics over time, as previously described [31,32]. The compound was added to 96-well microplates containing SDB plus fungal inoculum (2.5 × 10^3^ CFU/mL) to be at MIC, 2 × MIC and 4 × MIC. At selected time points (0, 1, 2, 4, 6, 8, 12 and 24 h), aliquots of the wells were seeded onto SDA plates and incubated at 37 °C for 24 h. The number of CFU/mL was determined and the data were transformed to log_10_. Sodium hypochlorite was used as a positive control, and an untreated group was included to monitor growth control for comparisons.

### 4.4. Mechanism(s) of Action of CAT

#### 4.4.1. Action on Cell Wall Biosynthesis

The MIC of CAT was determined by the Clinical & Laboratory Standards Institute (CLSI) microdilution technique [29] in the presence of an osmotic protector—sorbitol (INLAB, São Paulo, Brazil), in order to check whether CAT acts on the cell wall of *C. albicans* (ATCC 60193 and CBS 562). Fungal inoculum was added to a final concentration of 2.5 × 10^3^ CFU/mL previously supplemented with sorbitol (0.8 M final concentration). Caspofungin diacetate (Sigma-Aldrich) was used as a positive control in this assay from an initial concentration of 0.004 mmol/L [33,34] due to its known action on the enzyme (1→3)-β-d-glucan synthase, which disturbs the integrity of the fungal cell wall. Sodium hypochlorite was also included at an initial concentration of 33.58 mmol/L. The plates were incubated at 37 °C and read after 24 h and 48 h [32,35,36].

#### 4.4.2. Action on Cell Membrane Permeability

In order to check whether CAT molecules complex with membrane ergosterol, the MIC of CAT was determined against *C. albicans* ATCC 60193 and CBS 562 as previously described by the CLSI (CLSI, 2002) in the presence of increasing concentrations of exogenous ergosterol (Sigma-Aldrich) (100, 200 and 400 µg/mL). Nystatin, which highly binds to ergosterol, was used as positive control [37,38] and a group treated with sodium hypochlorite was also included. A negative control was also prepared with 96% ethanol and Tween 80, which were used in the preparation of ergosterol solutions. The plates were incubated at 37 °C for 24–48 h [35].

### 4.5. Effects of CAT on Fungal Micromorphology

The effects of CAT against blastoconidia, pseudohyphae and chlamydospores of *C. albicans* CBS 562 were determined through a microculture assay using cornmeal agar (CA, HiMedia Laboratories, Mumbai, India) plus Tween 80 [39]. CAT was added to CA before solidification at MIC_75_ and higher concentrations. Then 1 mL of CA plus CAT was placed on a glass slide and two parallel grooves were made after medium solidification using sterile disposable straps containing the tested strain. A glass coverslip was placed over the medium and the slides were placed in a moist chamber at 37 °C for 48 h. Nystatin and sodium hypochlorite were used as positive controls. The development and absence of typical structures such as blastoconidia, pseudohyphae and chlamydospores was observed by light microscopy (40× magnification).

### 4.6. Effects of CAT on C. Albicans Biofilms

We next investigated the anti-biofilm potential of CAT against *C. albicans* ATCC 60193 at different concentrations (MIC, 2 × MIC and 4 × MIC) and administration time points (on early adhesion and during/after biofilm formation). Treatments were carried out in a way as to simulate two possible clinical applications: a short-contact period of 1 min every 8 h, simulating immersion of the denture into the antimicrobial solution during daily oral hygiene; or a prolonged-contact period for 8 consecutive hours, simulating denture soaking overnight. Sodium hypochlorite was used as the positive control at different concentrations as well (MIC, 2 × MIC and 4 × MIC). Medium sterility and untreated growth controls were also included in all assays. The percentage of biofilm inhibition (I%) was calculated as compared to the untreated group, which represents 100% biofilm formation. The data were categorized into scores for further statistical analysis: a score of 1 reflected I% ≤ 25%; a score 2 indicated 25% < I% ≤ 50%; a score of 3 indicated 50% < I% ≤ 75%; and a score of 4 indicated 75% < I% ≤ 100%.

#### 4.6.1. Effects on Early Biofilm Adherence

First, *C. albicans* ATCC 60193 inoculum (2.5 × 10^5^ CFU/mL) was added to the wells of 96-well microplates along with CAT at different concentrations (MIC, 2 × MIC and 4 × MIC). The plate was incubated for 2 h at 37 °C to allow for initial adherence. Then the wells were washed with PBS in order to remove unbound cells; fresh SDB was added, and the plates were incubated again for 48 h at 37 °C. For biofilm quantification, the wells were washed twice with PBS, air-dried for 45 min, dyed with 0.4% crystal violet and unstained with 95% EtOH. Absorbance values were read at 600 nm using a microplate reader (GloMax-Multi, PROMEGA, Madison, WI, USA) [40].

#### 4.6.2. Effects on Mature Biofilm Formation

Fungal inoculum was added to 96-well plates and incubated for 2 h at 37 °C for initial adherence. The wells were washed with PBS and adhered cells were treated with CAT at MIC, 2 × MIC and 4 × MIC for 1 min or 8 consecutive hours. Then the wells were washed again with PBS and remained with fresh SDB for 48 h at 37 °C. Biofilm was quantified as described above, and the percentage of inhibition was calculated based on the untreated growth control.

#### 4.6.3. Reduction of Preformed Mature Biofilm

The procedures previously described for early adherence were followed. Then the wells were washed with PBS to remove unbound cells; SDB was added, and the plates were incubated at 37 °C for 48 h to allow mature biofilm formation. Subsequently, the medium was aspirated and the wells were washed with PBS to remove planktonic cells. CAT was added at MIC, 2 × MIC and 4 × MIC for 1 min or 8 consecutive hours, then aspirated and replaced by fresh culture medium. The plates were incubated at 37 °C. This procedure was repeated every 8 h for 1 and 2 days, totaling three- and six 1-minute applications; or once a day, totaling one- and two-8-hour applications, respectively. Biofilm cells were quantified as previously described.

### 4.7. Cytotoxic Effects of CAT on Human Erythrocytes

Peripheral blood samples were collected from five healthy individuals aged 18 to 40 years, with no history of hemoglobin or erythrocyte disorders among first-degree relatives. The blood samples were centrifuged for separation of plasma, platelets, white and red cells [41]. Erythrocytes were washed in PBS and then diluted to a 2% solution in PBS. A 1:1 suspension of erythrocytes and CAT at different concentrations (ranging from 88.75 to 2.77 mmol/L) was dispensed into the wells of 96-well U-bottom plates for 1 h. Then the supernatant of each well was collected and transferred to a 96-well flat-bottom plate to measure the hemolytic activity based on absorbance at 560 nm (Multi-GloMax reader, PROMEGA, Madison, WI, USA). Distilled water, sodium hypochlorite (0.5%) and saline were used as controls. The percentage of hemolysis was calculated using the formula: (AS − AN/AP − AN) × 100; where, AS, AN, and AP correspond to the absorbance of the test substance, negative control and positive control, respectively. All procedures were performed in accordance with the ethical standards of the institutional and national research committee and with the Helsinki declaration. This study was previously approved by the review institutional board of the Center for Health Sciences at the Federal University of Paraiba (protocol CAAE: 43914615.0.0000.5188).

### 4.8. Statistical Analysis

All tests were performed in triplicate of independent experiments and analyzed on IBM SPSS Statistical for Windows (version 20.0, IBM Corp., Armonk, NY, USA, 2011). The growth kinetics data were checked for normality using the Shapiro-Wilk’s test and analyzed by one-way analysis of variance (ANOVA) with Tukey’s post-test (*p* ≤ 0.05). The biofilm data were analyzed using Mann-Whitney and Kruskal-Wallis tests for comparisons between the groups and CAT concentrations (*p* ≤ 0.05).

## Figures and Tables

**Table 1:** Antifungal activity of chloramine T (CAT), sodium hypochlorite (NaOCl) and nystatin against *Candida* spp. strains. Values are expressed in mmol/L.

Strain	CAT			NaOCl			Nystatin		
	MIC	MFC	MFC/MIC Ratio	MIC	MFC	MFC/MIC Ratio	MIC	MFC	MFC/MIC Ratio
*Candida albicans* ATCC 60193	2.77	5.54	2 (Fungicidal)	8.39	8.39	1 (Fungicidal)	0.0004	0.0008	2 (Fungicidal)
*Candida albicans* CBS 562	0.69	2.77	4 (Fungistatic)	4.19	8.39	2 (Fungicidal)	0.0004	0.0008	2 (Fungicidal)
*Candida tropicalis* ATCC 750	2.77	2.77	1 (Fungicidal)	8.39	8.39	1 (Fungicidal)	0.0004	0.0004	1 (Fungicidal)
*Candida tropicalis* CBS 94	5.54	5.54	1 (Fungicidal)	16.79	16.79	1 (Fungicidal)	0.0004	0.0004	1 (Fungicidal)
*Candida krusei* ATCC 3413	1.38	1.38	1 (Fungicidal)	4.19	4.19	1 (Fungicidal)	0.001	0.001	1 (Fungicidal)
*Candida krusei* CBS 73	1.38	1.38	1 (Fungicidal)	4.19	4.19	1 (Fungicidal)	0.0008	0.0008	1 (Fungicidal)
*Candida glabrata* IZ 07	5.54	11.09	2 (Fungicidal)	16.79	16.79	1 (Fungicidal)	0.0004	0.0008	2 (Fungicidal)

**Table 2:** Effect of an osmotic protector (sorbitol) on the MIC of chloramine T (CAT), sodium hypochlorite (NaOCl) and caspofungin against *C. albicans* strains.

Strain	MIC (mmol/L)					
	CAT		NaOCl		Caspofungin	
	Without Sorbitol	With Sorbitol	Without Sorbitol	With Sorbitol	Without Sorbitol	With Sorbitol
*Candida albicans* ATCC 60193	2.77	22.18	8.39	33.58	<0.0003	>0.0045
*Candida albicans* CBS 562	0.69	11.09	4.19	33.58	<0.0003	0.0045

**Table 3:** Effects of different concentrations of exogenous ergosterol on the MIC of chloramine T (CAT), sodium hypochlorite (NaOCl) and nystatin against *C. albicans* strains. Values are expressed in mmol/L.

	CAT				NaOCl				Nystatin			
	Absence of Ergosterol	Presence of Ergosterol			Absence of Ergosterol	Presence of Ergosterol			Absence of Ergosterol	Presence of Ergosterol		
		100 µg/mL	200 µg/mL	400 µg/mL		100 µg/mL	200 µg/mL	400 µg/mL		100 µg/mL	200 µg/mL	400 µg/mL
*Candida albicans* ATCC 60193	2.77	5.54	5.54	5.54	8.39	16.79	16.79	16.79	0.0004	0.001	0.003	0.003
*Candida albicans* CBS 562	0.69	5.54	5.54	5.54	4.19	16.79	33.58	33.58	0.0004	0.001	0.001	0.001

**Table 4:** Percentage of inhibition (%I) of adherence and mature biofilm formation of *Candida albicans* ATCC 60193 treated with chloramine T (CAT) or sodium hypochlorite (NaOCl) with different exposure times.

Concentration	Inhibition of Initial Adherence		Inhibition Of Mature Biofilm Formation			
	Group 1 (Exposure time: 2 h)		Group 2 (Exposure time: 1 min)		Group 3 (Exposure time: 8 h)	
	CAT	NaOCl	CAT	NaOCl	CAT	NaOCl
MIC	25% < %I ≤ 50% ^Aa^	25% < %I ≤ 50% ^Aa^	25% < %I ≤ 50% ^Aa^	25% < %I ≤ 50% ^Aa^	%I ≤ 25% ^Aa^	25% < %I ≤ 50% ^Aa^
2 × MIC	%I ≤ 25% ^Aa^	50% < %I ≤ 75% ^Ba^	25% < %I ≤ 50% ^Aa^	25% < %I ≤ 50% ^Aa^	25% < %I ≤ 50% ^Aa^	25% < %I ≤ 50% ^Aa^
4 × MIC	25% < %I ≤ 50% ^Aa^	25% < %I ≤ 50% ^Aa^	25% < %I ≤ 50% ^Aa^	25% < %I ≤ 50% ^Aa^	25% < %I ≤ 50% ^Aa^	25% < %I ≤ 50% ^Aa^

Different uppercase letters in the lines represent a significant difference between the substances in each group (Mann-Whitney test, *p <* 0.05). Equal lowercase letters in each column represent similarity between the concentrations of each substance (Kruskal-Wallis test, *p <* 0.05).

**Table 5:** Percentage of inhibition (%I) of preformed biofilms of *Candida albicans* ATCC 60193 treated with chloramine T (CAT) or sodium hypochlorite (NaOCl) with different exposure times.

Concentration	Group 4 (Exposure time: 3 × 1 min)		Group 5 (Exposure Time: 6 × 1 min)		Group 6 (Exposure time: 8 h)		Group 7 (Exposure time: 2 × 8 h)	
	CAT	NaOCl	CAT	NaOCl	CAT	NaOCl	CAT	NaOCl
MIC	50% < %I ≤ 75% ^Aa^	%I ≤ 25% ^Aa^	%I ≤ 25% ^Aa^	%I ≤ 25% ^Aa^	50% < %I ≤ 75% ^Aa^	50% < %I ≤ 75% ^Aa^	25% < %I ≤ 50% ^Aa^	25% < %I ≤ 50% ^Aa^
2 × MIC	50% < %I ≤ 75% ^Aa^	%I ≤ 25% ^Ba^	%I ≤ 25% ^Aa^	%I ≤ 25% ^Aa^	50% < %I ≤ 75% ^Aa^	25% < %I ≤ 50% ^Aa^	25% < %I ≤ 50% ^Aa^	25% < %I ≤ 50% ^Aa^
4 × MIC	50%< %I ≤ 75% ^Aa^	%I ≤ 50% ^Aa^	25% <%I ≤ 50% ^Aa^	%I ≤ 25% ^Ba^	50% < %I ≤ 75% ^Aa^	25% < %I ≤ 50% ^Aa^	25% < %I ≤ 50% ^Aa^	25% < %I ≤ 50% ^Aa^

Different uppercase letters in the lines represent a significant difference between the substances in each group (Kruskal-Wallis test, *p* < 0.05). Equal lowercase letters in each column represent similarity between the concentrations of each substance (Mann-Whitney test, *p* < 0.05).
